# Age-period-cohort analysis of low birth weight, early-term delivery, induction, and cesarean-section, US births 1990-2019

**DOI:** 10.1093/aje/kwag147

**Published:** 2026-06-24

**Authors:** Chloe A Teasdale, Sean Locke, Zachary Shahn, Saskia Rueda, Elizabeth A Kelly, Francisco Bolumar, Heidi E Jones

**Affiliations:** Department of Epidemiology and Biostatistics, City University of New York (CUNY), Graduate School of Public Health & Health Policy (SPH), New York, NY, United States; Institute for Implementation Science in Population Health (ISPH), CUNY SPH, New York, NY, United States; Department of Epidemiology and Biostatistics, City University of New York (CUNY), Graduate School of Public Health & Health Policy (SPH), New York, NY, United States; Department of Epidemiology and Biostatistics, City University of New York (CUNY), Graduate School of Public Health & Health Policy (SPH), New York, NY, United States; Institute for Implementation Science in Population Health (ISPH), CUNY SPH, New York, NY, United States; Department of Epidemiology and Biostatistics, City University of New York (CUNY), Graduate School of Public Health & Health Policy (SPH), New York, NY, United States; Department of Obstetrics and Gynecology, University of Cincinnati College of Medicine, Cincinnati, OH, United States; Department of Epidemiology and Biostatistics, City University of New York (CUNY), Graduate School of Public Health & Health Policy (SPH), New York, NY, United States; Department of Public Health Sciences, University of Alcalá, Alcalá de Henares, Madrid, Spain; Department of Epidemiology and Biostatistics, City University of New York (CUNY), Graduate School of Public Health & Health Policy (SPH), New York, NY, United States; Institute for Implementation Science in Population Health (ISPH), CUNY SPH, New York, NY, United States

**Keywords:** low birth weight, early-term delivery, induction, c-section, maternal age, age-period-cohort, APC, cohort effects

## Abstract

We conducted an age-period-cohort (APC) analysis to distinguish age, period, and cohort effects underlying temporal trends in low birth weight (LBW: <2500 g), early-term deliveries (ETD: 37-38 weeks), induction, and cesarean-section (c-section) using US birth records from 1994-2019 for all singleton, first, full-term (37-44 weeks gestation) births (*n* = 8 194 523). Using the APC-interaction method, we estimated age, period, and cohort effects as odds ratios with 95% CI. Age and period effects were observed for all outcomes. Pregnant women/people aged 40-44 years had increased odds of LBW (OR 1.23, 95% CI, 1.19-1.26), ETD (OR 1.24, 95% CI, 1.23-1.26), induction (OR 1.05, 95% CI, 1.04-1.06), and c-section (OR 2.28, 95% CI, 2.26-2.30) compared to the total population. Odds of LBW (OR 1.14, 95% CI, 1.13-1.16), ETD (OR 1.11, 95% CI, 1.10-1.12), and induction (OR 1.71, 95%,CI, 1.70-1.72) were higher in 2019 compared to the study period average. Cohort effects not explained by age and period effects were observed for women aged 40-44 years in 2019 indicated by higher odds of ETD (OR 1.08, 95% CI, 1.05-1.10), induction (1.10, 95% CI, 1.07-1.12), and c-section (OR 1.09, 95% CI, 1.06-1.11). These findings suggest obstetric management of pregnant women ≥40 years changed in recent decades in ways not expected based on age and temporal trends alone.

## Introduction

Birth weight in the United States rose steadily over the 20th century but began a roughly 25-year decline starting in the 1990s.^1^ While the drop in birth weight among US infants, from 3316 g in 1990 to 3248 g in 2013 (where it has remained roughly the same since), is relatively modest, the fact that birth weight decreased is concerning.^2^ There may be multiple factors contributing to declining birth weight, including increases in infants born at low birth weight (LBW) (<2500 g) and preterm deliveries (<37 weeks gestation); however, these may not explain all of the decrease. Over the period starting in the 1990s to the present, there has been a small increase in the proportion of infants born at LBW (from 7% to 9% of all US births).^3^^,^^4^ The leading cause of LBW, preterm delivery, also increased during this period,^4^^,^^5^ while the proportion of births with macrosomia (>4000 g) declined from 8.9% in 2004 to 7.4% in 2022.^6^

Health risks from LBW, very low birth weight (VLBW) (<1500 g), and preterm birth are significant and can have lasting effects into adulthood.^7-9^ VLBW is associated with infection risk, respiratory and cardiovascular complications, cerebral palsy, and mortality.^7^ Compared to normal-weight infants, those born at 1500-2500 g are more likely to have stunted growth, developmental delays, and develop diabetes and hypertension.^10^^,^^11^ Rising prevalence of hypertension and heart disease in women of reproductive age in recent decades, along with more pregnancies in women >35 years, have contributed to risk of LBW and preterm birth.^7^^,^^9^^,^^12^^,^^13^ Emerging evidence also suggests that older paternal age and environmental factors, including exposure to air pollution, toxins, and warming temperatures, may also affect gestational age and weight at birth.^14-16^

Preterm births do not account for all of the decline in average birth weight however, as evidenced by lower average weight among full-term normal-weight (≥2500 g) infants.^3^^,^^17^ “Early-term” deliveries (ETD), those occurring at 37-38 weeks gestation both naturally and via induction and/or cesarean-section (c-section), may be contributing to smaller full-term normal-weight infants. From 2014 to 2023, the proportion of ETD among all full-term births increased from 24% to 30%, while births occurring at 40 weeks decreased from 22% to 18%.^5^ Birth at earlier gestational age among full-term infants is associated with increased postnatal mortality, poorer academic achievement in elementary school, impaired lung capacity in school-age children and adolescents, and higher mortality in young adults.^18-20^ A US study of births from 1995-2009, found an 86% increase in deliveries at 37-38 weeks gestation among full-term infants with no indication of medical need for early delivery, and showed that infants born earlier experienced more respiratory distress and need for ventilation.^21^

Over the period when US birth weight plateaued, there was a shift in obstetric practices that may have influenced gestational age at delivery and birth weight. Between 1990 and 2023, the proportion of singleton first deliveries in the United States that were induced increased from 12% to 32%.^4^^,^^22^^,^^23^ The proportion of c-section deliveries also rose, from 25% of all births in 1990 to 32% in 2023. Among singleton, full-term, nulliparous, head first deliveries, which are termed “lower risk,” 27% were by c-section in 2023 in the United States.^4^^,^^23^ Increased use of nonmedically indicated induction and c-section may be contributing to earlier delivery and, in turn, to lower overall birth weight in the United States.

Given temporal trends in obstetric practices and in maternal age, we conducted an age-period-cohort (APC) analysis for the period 1990-2019 to disentangle drivers of changes in the proportions of full-term singleton first US births that were classified as LBW, ETD, and that involved induction or c-section. APC studies are a type of statistical analysis used to measure 3 types of effects that impact temporal trends in outcomes: those related to the aging process, period effects (caused by events or changes at a specific point in time that impact the entire population), and cohort effects which represent the life-course trajectory of individuals born during a specific time period, and can be conceptualized as the interaction between age and period effects.^24^ While both increased maternal age at first birth and period effects, such as changes in obstetric practices, have been identified as important drivers of temporal changes in birth outcomes, no APC analyses have been conducted to identify whether changes occurring over time have differential effects on specific age groups.

## Methods

We used anonymized datasets of birth records from the US National Vital Statistics System, which includes demographic characteristics of pregnant women and other pregnant people (hereafter pregnant women). Our analysis includes all singleton, first-born infants born between 37- and 44-weeks gestation to women and people who can become pregnant aged 15-44 years residing in all 50 states. We excluded multiple births due to higher likelihood of LBW and preterm delivery,^17^ infants born to women <15 years or older than 44 years (due to small sample sizes), and records missing gestational age, birth weight, and maternal age. We restricted to first-born infants (to understand how changes in mean birth weight are a result of later maternal age) and to full-term births based on recorded gestational age at delivery in weeks based on last menstrual period (LMP). The obstetric estimate of gestation at delivery (OE), which incorporates information from the medical record including early ultrasound dating, is now widely used for gestational dating; however, it was not adopted in all states until 2007, while LMP has been reported since 1981.^25^ As our study includes data from 1994 through 2019, gestational age at delivery was estimated using LMP (without OE) to ensure consistency in dating across the entire period and, importantly, to avoid mistaking changes in gestational dating for period effects.

Maternal age at delivery was examined as 5-year groupings: 15-19, 20-24, 25-29, 30-34, 35-39, and 40-44. To avoid overlapping cohorts, we looked at periods using 1 year of data: 1994, 1999, 2004, 2009, 2014, and 2019 to represent the periods of interest. Maternal cohorts represent the birth year of the pregnant women and were estimated based on maternal age at delivery and year of delivery: 1960-1964, 1965-1969, 1970-1974, 1975-1979, 1980-1984, 1985-1989, 1990-1994, 1995-1999, and 2000-2004. We did not include birth record data from the most recent period (2020-2024), as 2024 birth data were unavailable.

### Outcomes

The analysis includes four outcomes measured as proportion of all first, singleton, full-term infants^1^: LBW (<2500 g),^2^ early-term (37-38 weeks gestation),^3^ induced (ie, induction), and^4^ c-section. Outcome data from birth certificates included birth weight in grams, gestational age at delivery based on LMP, and indication of induction and delivery by c-section.

### Statistical analysis

We conducted APC analyses according to the age-period-cohort-interaction (APC-I) methods developed by Luo and Hodges.^24^ Similar to other APC approaches, this method conceives of age and period effects as main effects (ie, differences between the overall average outcome and average outcomes in each age or period group), and it estimates cohort effects as the interaction between age and period effects (ie, for each specific age and period, the difference between the observed average outcome observed for that age group in that period and what would be predicted based on age and period effects alone). We selected the APC-I methodology for its ability to addresses the “identification” problem inherent to other approaches to APC analysis, which arises from the dependence of cohort effects on the age and period effects, which results in the inability to distinguish the 3 effects simultaneously (prior methods required holding either age or period constant). The APC-I model estimates age and period effects, while quantifying cohort effects as the dependent, interactive effects of age and period. This modeling strategy focuses on unique cohort differences that cannot be captured by age and period effects.

The following is the APC-I model:


\begin{equation*} g(E(Y_{ij}))=\mu +\alpha_{i}+\beta_{j}+\alpha\beta_{ij(k)}, \end{equation*}

where 𝜇 denotes the global outcome mean; *α_i_* denotes the main age effect associated with *i*th age category; ** β *_j_* denotes the main period effect associated with the *j*th period; *α* β **_*ij*(*k*)_, denotes the interaction of the *i*th age group and *j*th period group, which corresponds to the effect of the *k*th cohort; and *g* is a link function (inverse logit for the logistic models used). These deviations captured by the interactions indicate variances that cannot be explained by the age and period effects alone, and therefore are explained by maternal cohort membership (ie, unique effects to women born in the same 5-year period). Models were not adjusted for covariates.

This study used publicly available records and was not considered human subjects research. Analyses were conducted in R V.4.04.

## Results

### Age and period effects

Our analysis included 8 194 523 singleton, full-term, first births. We found age and period effects for all 4 outcomes. The highest proportions of births that were LBW were in women aged 15-19 years (4.0%) and 40-44 years (4.0%) across all periods, and lowest among women aged 25-29 years (2.6%). LBW increased over time for all age groups (Figure 1). In 1994, 3.0% of births were LBW compared to 3.5% in 2019. APC-I models indicate that women aged 15-19 years and 40-44 years had 1.25 (95% CI, 1.23-1.26) and 1.23 (95% CI, 1.19-1.26) times higher odds, respectively, of having LBW infants compared to the overall population. Women delivering in the most recent period, 2019, had 1.14 (95% CI, 1.13-1.16) times higher odds of having LBW infants compared to all periods (Table 1).

**Figure 1:**
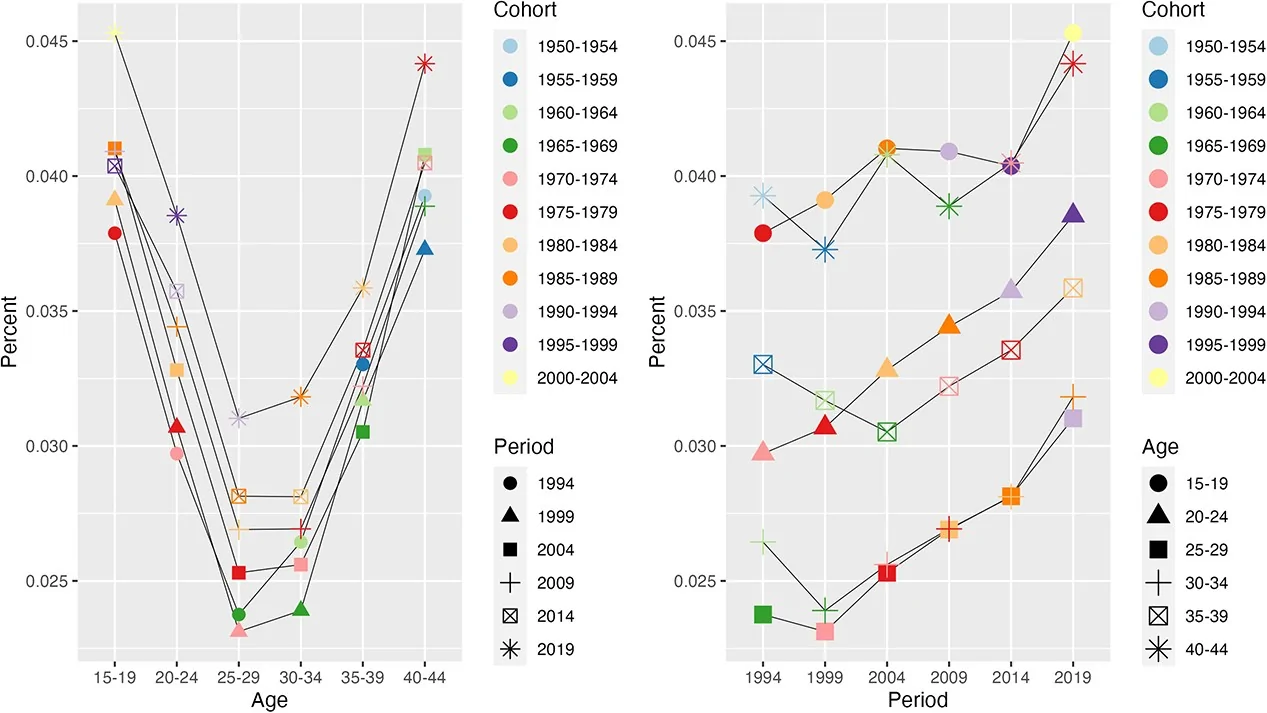
Proportion of low birth weight (<2500 g) among all singleton, full-term (≥37 weeks), first births in the US 1994-2019 by (A) age group and (B) period.

**Table 1:** Estimated age and period main effects for proportion of all singleton, full-term, first births in the US that are low birth weight, early-term delivery, induced delivery (induction), and c-section delivery (1994-2019) using the APC-I model with the total population as the reference group.

	**Low birth weight (<2500 g)**			**Early-term**			**Induction**			**C-section**		
	**OR**	**95% CI**	**Sig**	**OR**	**95% CI**	**Sig**	**OR**	**95% CI**	**Sig**	**OR**	**95% CI**	**Sig**
**Age**												
**15-19**	1.25	1.23-1.26	^b^	1.00	1.00-1.01	^a^	0.91	0.91-0.92	^b^	0.48	0.48-0.48	^b^
**20-24**	1.02	1.01-1.03	^b^	0.92	0.92-0.93	^b^	1.04	1.04-1.05	^b^	0.66	0.66-0.66	^b^
**25-29**	0.79	0.78-0.80	^b^	0.90	0.89-0.90	^b^	1.04	1.04-1.05	^b^	0.84	0.83-0.84	^b^
**30-34**	0.82	0.81-0.82	^b^	0.92	0.92-0.92	^b^	0.97	0.97-0.98	^b^	1.07	1.07-1.08	^b^
**35-39**	0.99	0.98-1.01	NA	1.05	1.04-1.05	^b^	0.98	0.98-0.99	^b^	1.55	1.54-1.56	^b^
**40-44**	1.23	1.19-1.26	^b^	1.24	1.23-1.26	^b^	1.05	1.04-1.06	^b^	2.28	2.26-2.30	^b^
**Period**												
**1994**	0.95	0.93-0.96	^b^	0.84	0.84-0.85	^b^	0.60	0.60-0.61	^b^	0.78	0.78-0.79	^b^
**1999**	0.92	0.91-0.93	^b^	0.97	0.96-0.97	^b^	0.84	0.83-0.84	^b^	0.79	0.79-0.80	^b^
**2004**	0.97	0.96-0.99	^b^	1.08	1.07-1.08	^b^	0.93	0.92-0.93	^b^	1.10	1.09-1.11	^b^
**2009**	1.00	0.99-1.02	NA	1.07	1.06-1.07	^b^	1.08	1.07-1.09	^b^	1.27	1.26-1.27	^b^
**2014**	1.03	1.02-1.05	^b^	0.96	0.96-0.97	^b^	1.16	1.15-1.17	^b^	1.11	1.10-1.12	^b^
**2019**	1.14	1.13-1.16	^b^	1.11	1.10-1.12	^b^	1.71	1.70-1.72	^b^	1.05	1.04-1.05	^b^

Abbreviations: APCI, age-period-cohort-interaction; NA, not applicable; OR, odds ratio; Sig, statistical significance.

a *P* < .01.

b *P* < .0001

Age and period effects were also observed for singleton, full-term, first births that were ETD, which was most common among women aged 40-44 years (31.3%) and least common in those 25-29 years (24.3%). ETD increased from 22.0% of births in 1994 to 26.8% in 2019 (Figure 2). In APC-I models, highest odds of ETD (compared to the overall population) were in women aged 40-44 (odds ratio [OR] 1.24, 95% CI, 1.23-1.26) and among deliveries in 2019 (OR 1.11, 95% 1.10-1.12) (Table 1).

**Figure 2:**
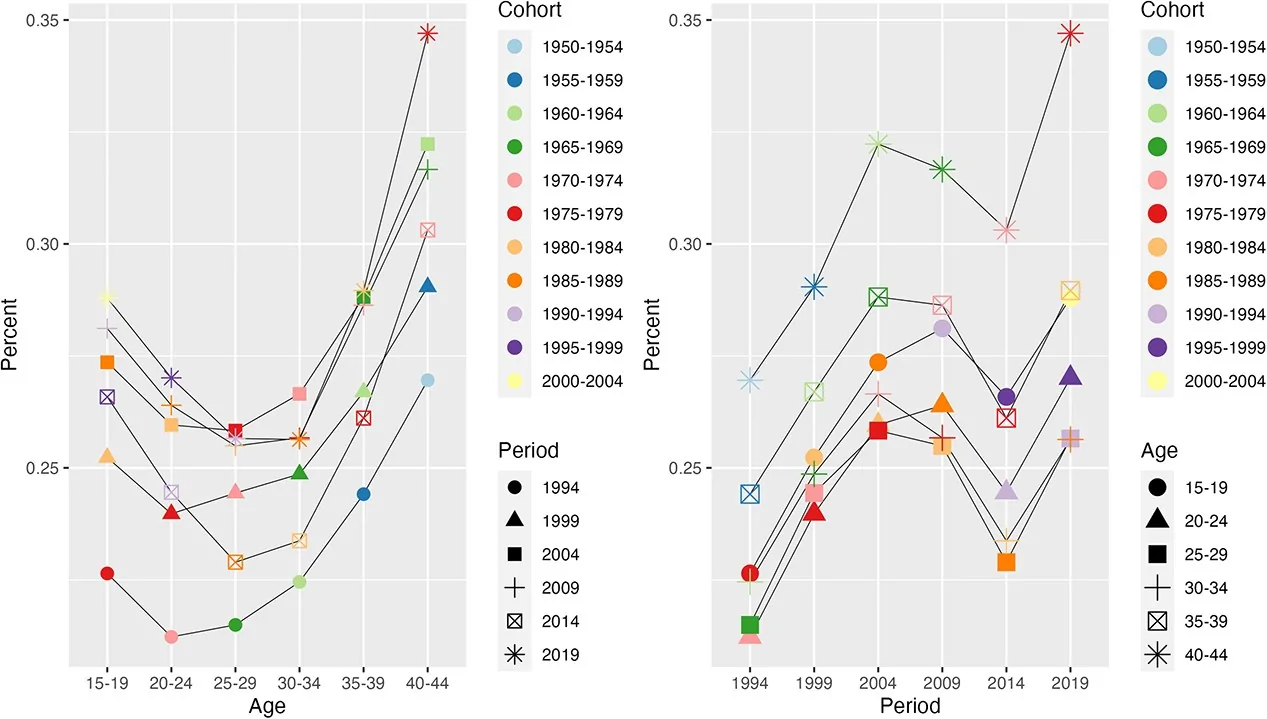
Proportion of early-term deliveries (37-38 weeks) among all singleton, full-term (≥37 weeks), first births in the US 1994-2019 (based on LMP gestational dating) by (A) age group and (B) period.

The proportion of births with induction increased with age; the highest proportion was in women aged 40-44 years (29.6%), and the lowest was in those aged 15-19 years (23.8%) (Figure 3). The proportion of births with induction increased over time in all age groups, from 16.9% in 1994 to 37.8% in 2019. Odds of induction were 71% higher in 2019 (OR 1.71, 95% CI, 1.70-1.72) compared to the average over the entire timeframe, and were slightly higher among women aged 40-44 (OR 1.05, 95% CI, 1.04-1.06) compared to the overall population (Table 1).

**Figure 3:**
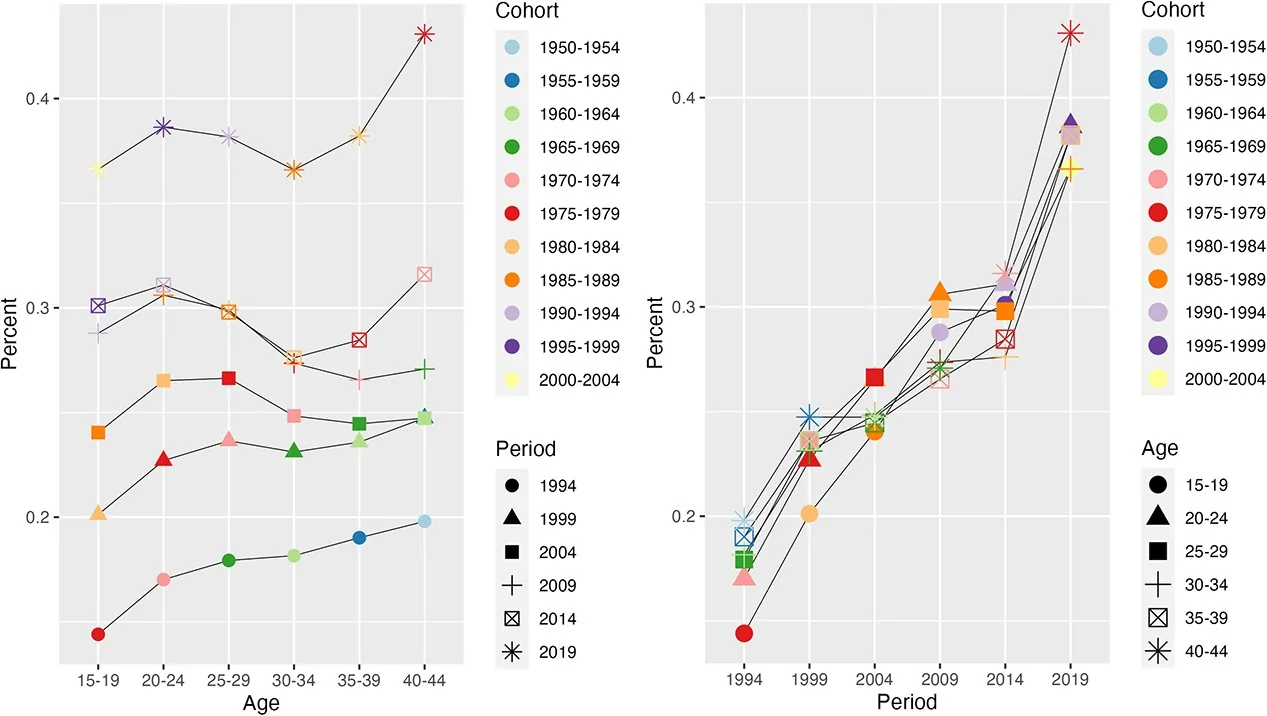
Proportion of births with induction among all singleton, full-term (≥37 weeks), first births in the US 1994-2019 by (A) age group and (B) period.

For c-sections, women aged 40-44 years (51.4%) had the highest proportion and 15-19-year-olds (17.5%) had the lowest (Figure 4A). C-section delivery of singleton, full-term, first births increased for all age groups from 21.6% in 1994 to a peak of 30.7% in 2009 (Figure 4). In the most recent period, 2019, 28.3% of births were by c-section. In APC-I models, highest odds of delivery by c-section (compared to the overall population) were in women aged 40-44 years (OR 2.28, 95% CI, 2.26-2.30) and in 2009 (OR 1.27, 95% CI, 1.26-1.27) (Table 1).

**Figure 4:**
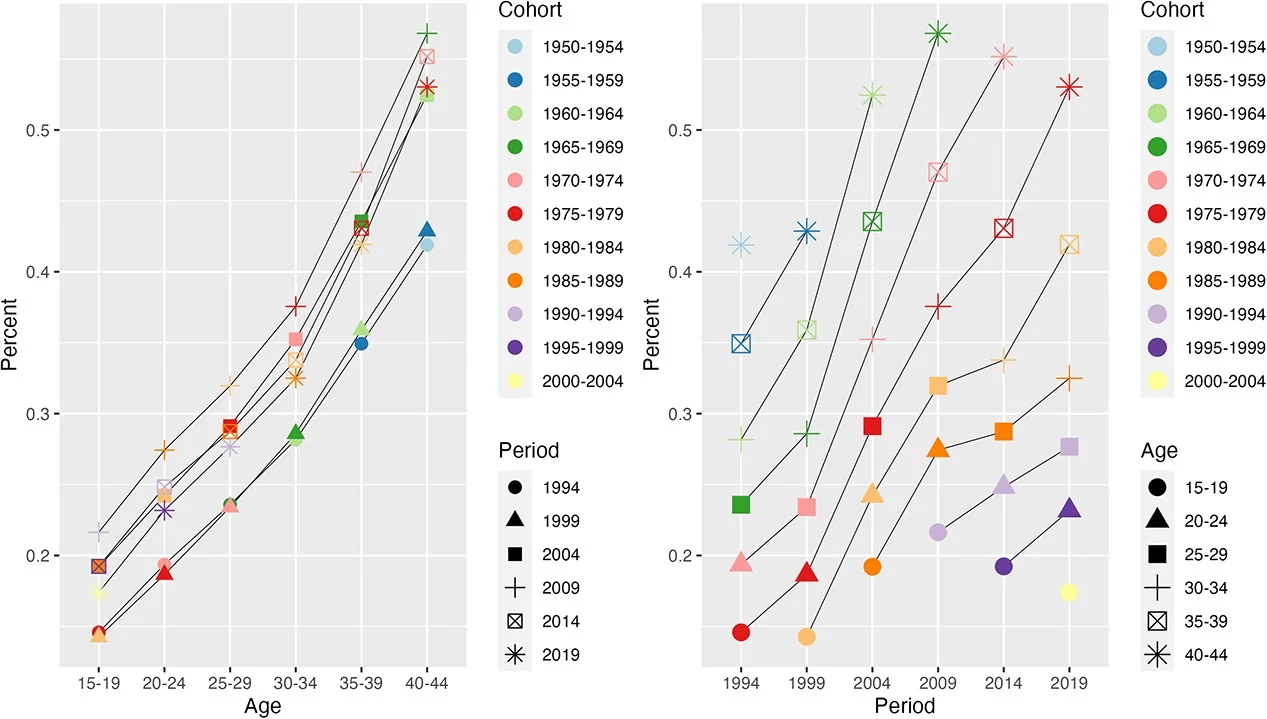
Proportion of births by c-section among all singleton, full-term (≥37 weeks), first births in the US 1994-2019 by (A) age group and (B) period.

### Cohort effects

Cohort effects, that is, effects not explained by the age and period trends alone, were examined both graphically and using interaction terms from the APC-I models for each outcome (Table 2). ORs for the cohort effects indicate deviations from the expected likelihood of the outcome based on age and period effects alone. While many of the cohort effects are statistically significant due to large sample size, we identified meaningful effects based on graphical representations and effect estimates (Table 2).

**Table 2:** Age-by-period interaction terms (representing cohort effects) for low birth weight (<2500 g), early-term delivery, induction, and c-section in the APC-I model including singleton, full-term first births in the US 1994-2019.

	**1994**			**1999**			**2004**			**2009**			**2014**			**2019**		
**Age**	**OR**	**95% CI**	**Sig**	**OR**	**95% CI**	**Sig**	**OR**	**95% CI**	**Sig**	**OR**	**95% CI**	**Sig**	**OR**	**95% CI**	**Sig**	**OR**	**95% CI**	**Sig**
**Low birth weight**																		
**15-19**	0.98	0.96-1.00	NA	1.04	1.02-1.06	^c^	1.04	1.02-1.06	^c^	1.00	0.98-1.02	NA	0.96	0.94-0.98	^c^	0.98	0.96-1.00	NA
**20-24**	0.93	0.91-0.95	^c^	0.99	0.97-1.01	NA	1.00	0.99-1.02	NA	1.03	1.01-1.05	^b^	1.03	1.01-1.05	^c^	1.01	0.99-1.03	NA
**25-29**	0.96	0.93-0.98	^c^	0.95	0.93-0.98	^c^	0.99	0.97-1.01	NA	1.03	1.00-1.05	^a^	1.04	1.02-1.06	^c^	1.04	1.02-1.06	^c^
**30-34**	1.03	1.01-1.06	^a^	0.96	0.93-0.98	^c^	0.97	0.95-0.99	^a^	1.00	0.97-1.02	NA	1.01	0.98-1.03	NA	1.04	1.02-1.06	^c^
**35-39**	1.07	1.03-1.10	^c^	1.05	1.02-1.09	^b^	0.95	0.93-0.99	^b^	0.98	0.95-1.01	NA	0.99	0.96-1.02	NA	0.96	0.94-0.99	^b^
**40-44**	1.04	0.96-1.11	NA	1.01	0.94-1.08	NA	1.05	0.99-1.11	NA	0.97	0.91-1.03	NA	0.98	0.92-1.03	NA	0.97	0.92-1.02	NA
**Early-term delivery**																		
**15-19**	0.97	0.96-0.98	^c^	0.97	0.96-0.98	^c^	0.98	0.97-0.98	^c^	1.02	1.01-1.03	^c^	1.05	1.04-1.06	^c^	1.02	1.01-1.03	^b^
**20-24**	0.97	0.96-0.98	^c^	0.99	0.98-1.00	^a^	0.99	0.98-1.00	^b^	1.02	1.01-1.03	^c^	1.02	1.01-1.03	^c^	1.01	1.00-1.02	^b^
**25-29**	1.01	1.00-1.02	^b^	1.04	1.03-1.05	^c^	1.01	1.00-1.05	^a^	1.00	0.99-1.01	NA	0.96	0.96-0.97	^c^	0.97	0.96-0.98	^c^
**30-34**	1.04	1.03-1.06	^c^	1.04	1.03-1.06	^c^	1.03	1.02-1.05	^c^	0.98	0.97-0.99	^c^	0.97	0.96-0.97	^c^	0.95	0.94-0.95	^c^
**35-39**	1.02	1.01-1.04	^b^	1.01	0.99-1.04	NA	1.00	0.99-1.02	NA	1.00	0.99-1.02	NA	0.98	0.97-0.99	^b^	0.98	0.97-0.99	^b^
**40-44**	0.98	0.95-1.02	NA	0.95	0.93-0.98	^c^	0.99	0.97-1.02	NA	0.98	0.95-1.00	NA	1.02	0.99-1.04	NA	1.08	1.05-1.10	^c^
**Induction**																		
**15-19**	0.85	0.84-0.86	^c^	0.92	0.91-0.93	^c^	1.02	1.01-1.03	^c^	1.11	1.11-1.12	^c^	1.11	1.11-1.12	^c^	1.01	1.00-1.02	NA
**20-24**	0.92	0.91-0.93	^c^	0.96	0.95-0.96	^c^	1.04	1.03-1.04	^c^	1.08	1.07-1.09	^c^	1.03	1.02-1.04	^c^	0.98	0.97-0.99	^c^
**25-29**	0.98	0.97-0.99	^c^	1.00	1.00-1.01	NA	1.04	1.03-1.05	^c^	1.04	1.04-1.05	^c^	0.97	0.96-0.98	^c^	0.96	0.95-0.97	^c^
**30-34**	1.07	1.06-1.08	^c^	1.04	1.03-1.05	^c^	1.01	1.01-1.02	^b^	0.99	0.98-1.00	^b^	0.94	0.93-0.95	^c^	0.96	0.95-0.97	^c^
**35-39**	1.11	1.09-1.12	^c^	1.05	1.03-1.06	^c^	0.97	0.96-0.99	^c^	0.93	0.92-0.94	^c^	0.95	0.94-0.97	^c^	1.00	0.99-1.01	NA
**40-44**	1.09	1.06-1.13	^c^	1.04	1.01-1.07	^b^	0.92	0.9-0.95	^c^	0.87	0.85-0.89	^c^	1.00	0.98-1.03	NA	1.10	1.07-1.12	^c^
**C-section**																		
**15-19**	1.02	1.01-1.03	^c^	1.00	0.99-1.01	NA	1.01	1.00-1.020	NA	1.01	1.00-1.02	^a^	1.01	1.00-1.02	NA	0.96	0.95-0.97	^c^
**20-24**	1.03	1.02-1.04	^c^	0.99	0.98-0.99	^b^	0.98	0.97-0.99	^c^	1.00	1.00-1.01	NA	1.01	1.00-1.01	NA	0.99	0.98-1.00	^a^
**25-29**	1.04	1.04-1.05	^c^	1.03	1.02-1.04	^c^	0.99	0.98-1.00	^b^	0.98	0.98-0.99	^c^	0.97	0.96-0.97	^c^	0.99	0.98-0.99	^c^
**30-34**	1.04	1.03-1.04	^c^	1.05	1.04-1.06	^c^	1.02	1.02-1.03	^c^	0.98	0.97-0.99	^c^	0.95	0.95-0.96	^c^	0.96	0.96-0.97	^c^
**35-39**	0.98	0.97-0.99	^b^	1.01	1.00-1.03	^a^	1.01	0.99-1.02	NA	1.00	0.99-1.02	NA	0.98	0.97-0.99	^b^	1.01	1.00-1.02	^a^
**40-44**	0.90	0.88-0.92	^c^	0.93	0.91-0.95	^c^	0.99	0.97-1.01	NA	1.02	1.00-1.04	NA	1.09	1.06-1.11	^c^	1.09	1.07-1.11	^c^

Abbreviations: APC-I, age-period-cohort-interaction; NA, not applicable; OR, odds ratio; Sig, statistical significance.

a *P* < .01.

b *P* < .001.

c *P* < .0001.

Period was differentially associated with the likelihood of ETD in different age groups, as illustrated in Figure 2. For all age groups, there was a steady increase from 1994 to 2009, followed by a slight decline in 2014, followed by an uptick in 2019. While the general pattern was similar across age groups, from 2014 to 2019, women aged 40-44 years had a much steeper increase relative to other age groups. This effect is also discernible from the cohort effect estimate for the interaction between age group 40-44 and period 2015-2019 (OR 1.08, 95% CI, 1.05-1.10) (Table 2).

Examining Figure 3, we see that age was differentially associated with the likelihood of induction in different periods, indicating that the decision to induce appears to have changed significantly over time. In the 1994 and 1999 periods, there was a steady and gradual increase in probability of induction as age increased. In 2004 and 2009, probability of induction peaked at ages 20-29 before declining. In the periods ending in 2014 and 2019, probability of induction was lowest at age 30-34 before sharply increasing for age group 35-39 and even more for age group 40-44. This pattern can also be observed in Figure 3B, as the slope for age group 40-44 over the periods 2014 and 2019 is much more steeply increasing than for any other age group. These effects are further discernible from the model estimates showing an OR of 1.10 (95% CI, 1.07-1.12) for the interaction between age group 40-44 and the period ending in 2019.

Figure 4A shows that there were strong monotonic age effects for c-section at all time periods. However, in 2014 and 2019, the effect of being in age group 40-44 is even more pronounced than in earlier periods. This can be seen from Figure 4A in the form of a steeper age slope between 35-39 and 40-44 for the lines corresponding to periods ending in 2014 and 2019. These effects are also discernible from the model estimates for the interactions between age group 40-44 and the period ending in 2019 (OR 1.09 95% CI, 1.07-1.11). Thus, being 40-44 years appears to have played a greater role in the decision to perform a c-section in recent periods.

## Discussion

In this study of US birth records from singleton, full-term, first births, we found that over the past 30 years, women aged 40-44 years were most likely to have LBW infants, deliver before 39 weeks, and have births involving induction or c-sections. LBW increased over the period for all age groups which may be, in part, due to earlier term deliveries at 37-38 weeks, as well as increased use of induction and c-sections. A unique aspect of our analysis was estimation of cohort effects through which we identified markedly higher proportions of births involving induction and c-section among women ≥40 years in recent years, trends that cannot be explained by the independent overall effects of age or period. These findings are an indication that obstetric management of pregnant women at advanced maternal age has changed in recent decades (due to clinical practice, clinical characteristics of older pregnant women or both), and provides one potential explanation for the continued plateau in birth weight among US infants.

There are no other APC studies with which to compare our findings showing increases in LBW, ETD, induction, and c-sections over the past 30 years; however, they are consistent with prior reports using birth records, as well as cohort and modeling studies.^4^^,^^5^^,^^22^ The purpose of our analysis was to describe trends rather than to explore causal relationships; nonetheless, our results may provide insight into the lack of progress in reducing LBW among full-term infants over the past 3 decades. The shift toward earlier delivery within the full-term period may be playing a role, along with, and possibly as a result of, increased use of induction and, until very recently, c-section. While our analysis cannot elucidate these relationships and identify a direct link to lower birth weight, a modeling study using data from 1990 to 2013 found that use of induction and c-section increased at all gestational ages over the period, and the authors concluded that had these obstetric practices not increased, US birth weight would have increased rather than decreased during this period.^22^ There is also evidence that exposure to heatwaves also increases the risk of ETD, suggesting a potential effect of climate change over this period.^26^ Additional studies with more recent data are needed to help understand the causes and consequences of increasing ETD on the health of pregnant women and infants.

Existing evidence suggests short-term negative effects from earlier delivery among full-term infants, including higher infant mortality.^21^^,^^27^ Studies of long-term outcomes of infants born ETD are needed as well to measure its impact throughout childhood, adolescence, and adulthood. It is notable that among all US births in 2023, 32% were by c-section^4^ and in our analysis, almost 40% of singleton, full-term first births in 2019 involved induction, which represents a 70% increase in odds of induction since 1994. Among high income countries, the United States is one of several with high c-section rates, well above the 10%-15% recommended by the World Health Organization, and the global average of 21%.^28^ While these interventions are undoubtably lifesaving, the high rate of their use in the United States should be examined.

The trends of increased use of induction and c-section may, in part, be explained by increased prevalence of health conditions that contribute to higher risk pregnancies, including obesity, diabetes, and hypertensive disorders, which are most common in older women.^29^ It is also important to note that use of induction in the United States increased in recent years, from 25.1% of all births in 2017 to 33.2% in 2023, likely, in part, driven by the ARRIVE trial. The ARRIVE study (published in 2018) compared expectant management vs planned induction at 39-weeks among low-risk nulliparous women.^29^ The study reported no increased risk of adverse infant outcomes and no effect on c-section delivery in the planned induction at 39-weeks arm. Subsequent studies have demonstrated a significant increase in induction following the trial.^30^^,^^31^ One of these studies, a retrospective cohort comparing low risk nulliparous US births before and after the trial, found that deliveries before 39 weeks increased, as did adverse maternal and neonatal outcomes.^30^ Continued monitoring is needed to understand the impact of changes in obstetric management following the ARRIVE trial, including studies examining adverse outcomes at delivery and after.

A critical contribution of our study is examination of cohort effects, conceptualized in our analysis as interactions between age and period, which help us understand how a pregnant woman’s age influenced interventions and birth outcomes over time. In our analysis, we explored whether there were indications of cohort effects, and our findings suggest that shifts in obstetric practices over the period, including increased use of labor induction and c-sections, changed at different rates based on a woman's age at delivery. Specifically, we found that for those aged 40-44 years in 2019, the likelihood that they had ETD and induction increased compared to previous periods, which warrants further investigation. While clinical guidelines from the American College of Obstetrics and Gynecology recommend induction at 39 weeks for women of advanced maternal age to reduce likelihood for stillbirth,^31^ these recommendations, nor the findings from the ARRIVE trial, should have resulted in increased inductions before 39 weeks. Research is needed to identify the reasons for changes in obstetric management of pregnant women aged 40-44 years over the past decades and to document the impact these change may have on the health of women and their infants.

Our study has several key strengths, including use of the APC-I analysis approach, which allows for identification of cohort effects while avoiding the critical limitation of APC analyses, the identification problem (ie, that age and period fully identify cohort, and thus standard regression models cannot generate estimates for all three effects).^24^ Limitations include exclusion of the most recent period (2024) due to lack of available data. Future studies should extend our analysis to include this period to understand how the COVID-19 pandemic may have resulted in unique period, and possible cohort effects. In addition, an inherent limitation of APC analyses is lack of identification of causal factors. This approach is most helpful in identifying groups who have been impacted by changes in age and period effects, and to inform hypothesis generation for studies examining potential causes. In the case of our findings, we believe future studies should examine changes in obstetric management especially for women of advanced maternal age to understand the reasons for the increased use of inductions, as well as to understand how early-term delivery among full-term infants may be affecting birth weight.

## Conclusion

Using US birth records from 1994 through 2019, we identified important trends indicating increasing LBW, ETD, and induction and high, but decreasing, c-section delivery among singleton, full-term first births. Through use of the APC study design, we identified previously unreported cohort effects which show significantly higher likelihood of ETD, induction, and c-section among women ≥40 years since 2010. These findings suggest obstetric management of pregnant women aged 40 years and older has changed in recent years in ways not expected based on prior age trends and overall increased use of induction and c-sections; the extent to which this is being driven by changes in clinical characteristics and/or clinical practice warrants further investigation.
